# Case report: Foreign body aspiration requiring extracorporeal membrane oxygenation

**DOI:** 10.3389/fped.2023.1189722

**Published:** 2023-07-10

**Authors:** Dylan Ginter, K. Taneille Johnson, Oliver Venettacci, Rachel D. Vanderlaan, Elaine Gilfoyle, Haifa Mtaweh

**Affiliations:** ^1^ Department of Critical Care Medicine, The Hospital for Sick Children, Toronto, ON, Canada; ^2^ Division of Cardiovascular Surgery, Hospital for Sick Children, Toronto, ON, Canada

**Keywords:** foreign body, status asthmaticus, ECMO, case report, intubation, bronchoscopy

## Abstract

**Introduction:**

Foreign body aspiration is a common cause of respiratory distress in pediatrics, but the diagnosis can be challenging given aspirated objects are mostly radiolucent on chest radiographs and there is often no witnessed choking event. We present a case of a patient who was initially managed as severe status asthmaticus, requiring veno-arterial extracorporeal membrane oxygenation (VA-ECMO) for refractory hypercarbia and hypoxemia, but was later found to have bilateral bronchial foreign body aspiration. This case is unique in its severity of illness, diagnostic dilemma with findings suggesting a more common diagnosis of asthma, and use of ECMO as a bridge to diagnosis and recovery.

**Patient case:**

A previously healthy 2-year-old boy presented during peak viral season with a 3-day history of fever, cough, coryza, and increased work of breathing over the prior 24 h. There was no reported history of choking or aspiration. He was diagnosed with asthma and treated with bronchodilator therapy. Physical examination revealed pulsus paradoxus, severe work of breathing with bilateral wheeze, and at times a silent chest. Chest radiographs showed bilateral lung hyperinflation. Following a brief period of stability on maximum bronchodilator therapies and bilevel positive pressure support, the patient had a rapid deterioration requiring endotracheal intubation, with subsequent cannulation to VA-ECMO. A diagnostic flexible bronchoscopy was performed and demonstrated bilateral foreign bodies, peanuts, in the right bronchus intermedius and the left mainstem bronchus. Removal of the foreign bodies was done by rigid bronchoscopy facilitating rapid wean from VA-ECMO and decannulation within 24 h of foreign body removal.

**Conclusion:**

Foreign body aspiration should be suspected in all patients presenting with atypical history and physical examination findings, or in patients with suspected common diagnoses who do not progress as expected or deteriorate after a period of stability. Extracorporeal life support can be used as a bridge to diagnosis and recovery in patients with hemodynamic or respiratory instability.

## Background

Foreign body aspiration (FBA) is a common cause of both pediatric lower and upper airway obstruction and respiratory distress (1). FBA most often occurs in children under three years old with small organic materials such as nuts and seeds (2) but can also include toys or other small objects (3). The most common presentation of FBA is cough, choking, and dyspnea, but may also present with non-specific symptoms or no symptoms at all (2). Less than 10% of aspirated objects are radio-opaque on chest radiograph (4), therefore radiographic findings are predominantly nonspecific with findings of air trapping, air leak, or no abnormalities, rendering x-rays of low utility in diagnosing FBA (2). The gold standard diagnosis and treatment of FBA is via bronchoscopic visualization and removal (5).

The differential diagnosis of FBA is broad, including more common diagnoses of asthma and lower respiratory tract infections (4). The diagnosis is often delayed, taking longer than 24 h in 60% of cases (2). There are no validated clinical prediction models to identify pediatric patients with FBA (5), therefore, a high index of suspicion is needed for timely diagnosis, especially if the presentation and clinical evolution are atypical. To illustrate this diagnostic challenge, we present a case of a 2-year-old with bilateral mainstem bronchial FBA, initially treated as life-threatening asthma who required cannulation to veno-arterial extracorporeal membrane oxygenation (VA-ECMO) for ventilation-refractory hypercarbia and hypoxemia.

## Case description

The legal guardians have provided informed consent for the preparation and publication of this case report.

A previously healthy 2-year-old boy with speech delay presented to care with a four-day history of coryza, increased work of breathing, and progressive coughing, initially thought to have stridor. He was treated with an epinephrine nebulizer and oral dexamethasone, following which his symptoms progressed to bilateral expiratory wheezing. The patient was then treated with back-to-back dosing of salbutamol and ipratropium, followed by methylprednisolone and magnesium sulphate by intravenous (IV) route, started on heated humidified high flow nasal cannula (HFNC) at 2 L/kg/min, and transferred to the tertiary care hospital with a presumptive diagnosis of severe asthma.

**Figure 1:**
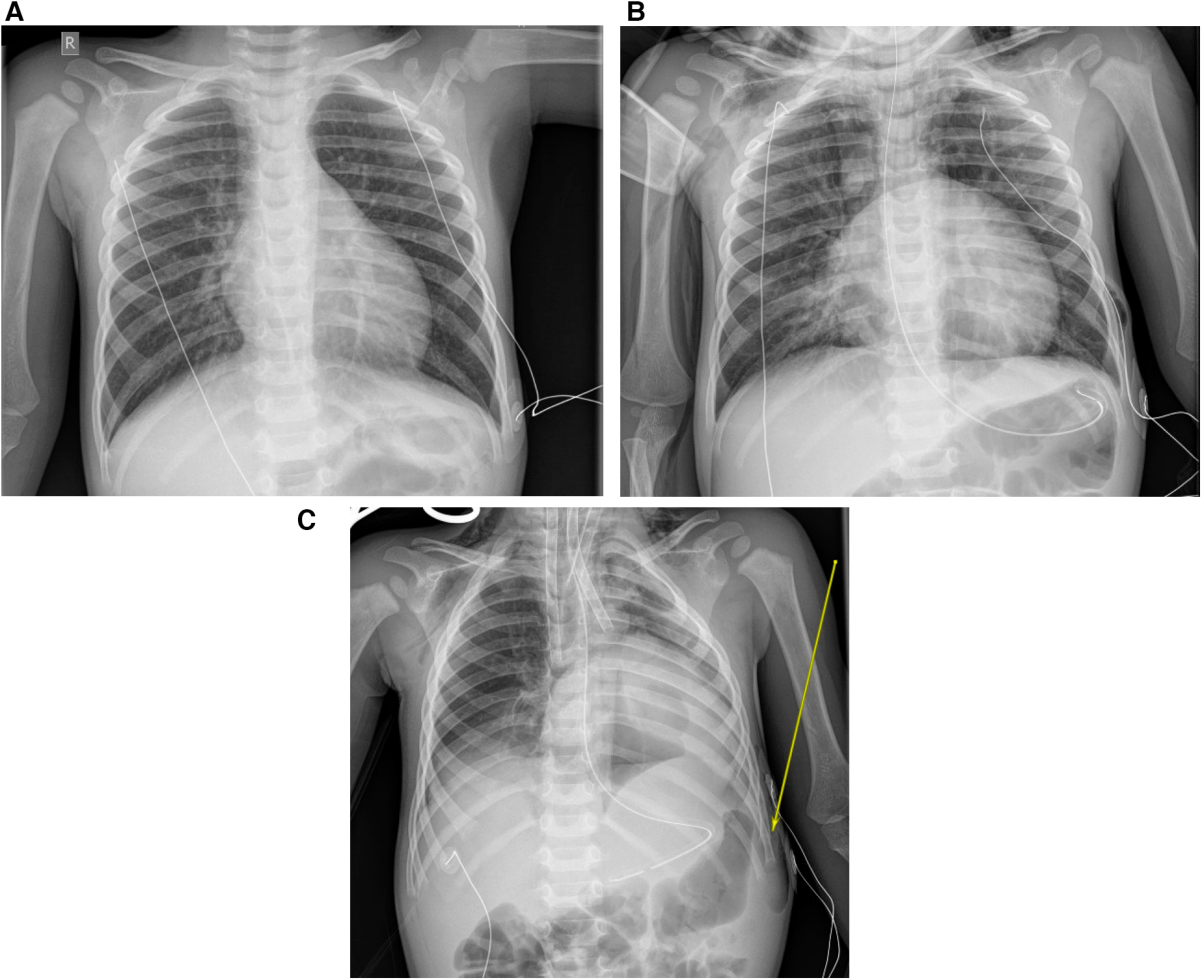
(**A**) Initial presentation chest x-ray. (**B**) Pre-intubation x-ray with complicated pneumomediastinum. (**C**) Post intubation and post-peripheral VA-ECMO cannulation.

Upon presentation to our care, he had severe work of breathing with bilateral wheeze and at times had a near silent chest, marked pulsus paradoxus, and an initial venous carbon dioxide (CO_2_) value of greater than 110 mmHg. Initial chest radiographs demonstrated bilateral hyperinflation, no consolidation, and no radio-opaque FB (Figure 1A). He was quickly escalated to bilevel positive airway pressure (BiPAP) support (inspiratory pressure 20 cmH_2_O, expiratory pressure 10 cmH_2_O) with continuous salbutamol nebulization and intravenous methylprednisolone. Given his severity of illness, the nebulized salbutamol was changed to an intravenous salbutamol infusion. Intravenous aminophylline and low dose ketamine infusions were also added for further bronchodilation.

**Figure 2:**
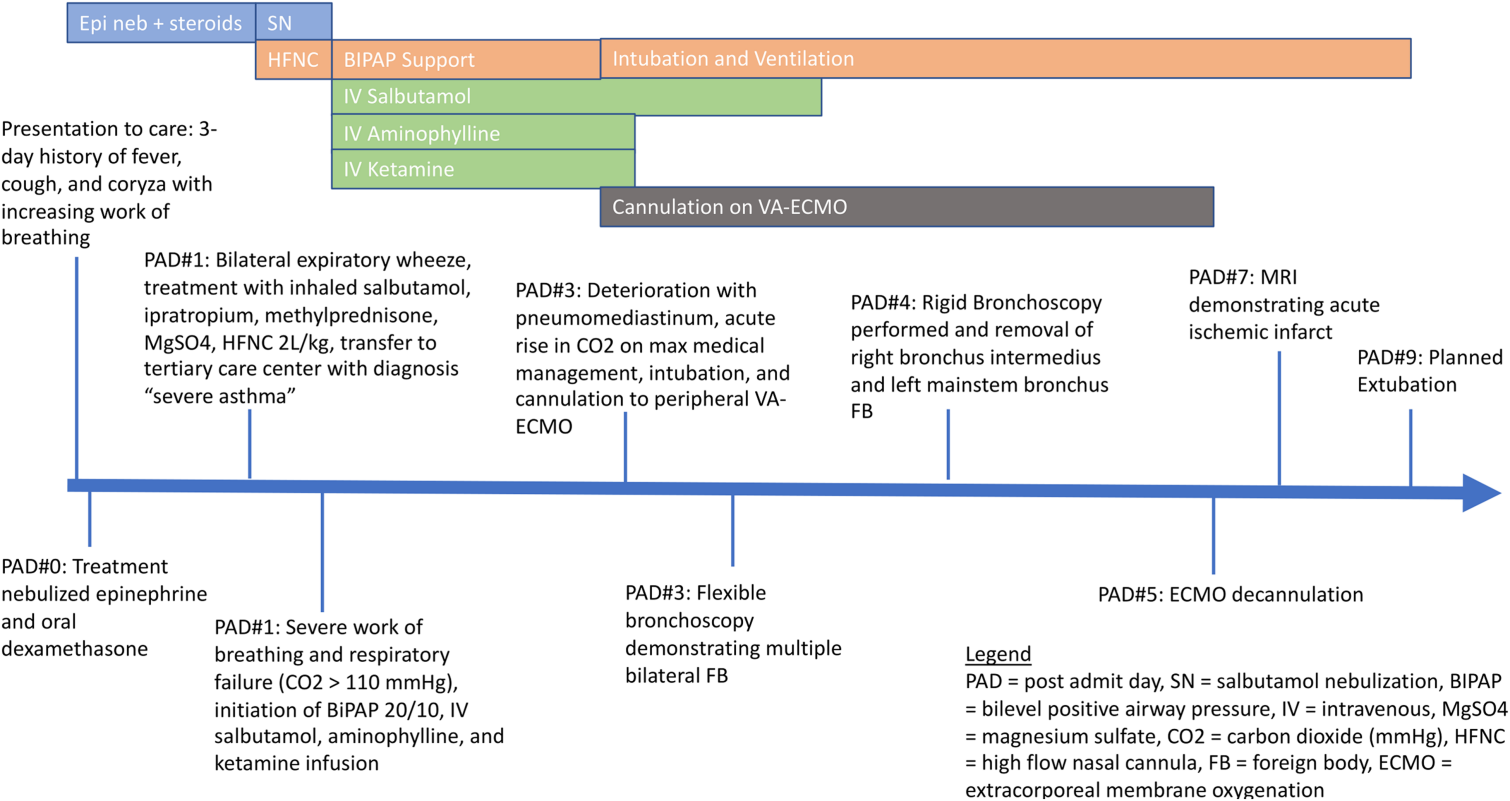
Pediatric intensive care unit timeline.

After a period of relative stability with reduction in the work of breathing, a normalization of arterial CO_2_ levels, and minimal oxygen requirement, he deteriorated on day 3 of his admission with development of a pneumomediastinum (Figure 1B) and an acute rise in arterial CO_2_. He was intubated then cannulated to peripheral VA-ECMO through the right common carotid artery and the right internal jugular vein, due to inadequate oxygenation and ventilation on conventional mechanical ventilation (Figure 1C). We performed a bedside flexible bronchoscopy shortly after cannulation to ECMO prompted by the unexplained deterioration after a period of improvement and that demonstrated multiple bilateral foreign bodies that were too large to aspirate. A rigid bronchoscopy successfully removed the foreign bodies, which were found to be peanuts, from the right intermediate bronchus and the left mainstem bronchus. Post-foreign body removal, the patient was weaned rapidly from bronchodilator therapy, VA-ECMO support and was decannulated on day 5 of admission. His VA-ECMO course was complicated by myocardial stun which improved post-decannulation, and findings on brain MRI suggestive of ischemic stroke, including acute infarcts in the right cortical and subcortical white matter and bilateral thalamic diffusion restriction. The patient was extubated on day 9 of admission and discharged from ICU shortly thereafter. At the time of manuscript preparation, the patient was diagnosed with autism spectrum disorder, which was suspected prior to his acute illness. His only current medication is enoxaparin for secondary stroke prophylaxis, and he is undergoing nutritional rehabilitation. Clinically he is well and regaining previously attained motor milestones (Figure 2).

## Discussion

This case demonstrates the diagnostic challenges of FBA when the history and physical exam are atypical; the nuances in clinical decision making in the acute stages of presentation of a patient with suspected severe status asthmaticus, where intubation might result in cardiopulmonary arrest, and how ECMO can be used as a bridge to diagnosis in the setting of severe refractory hypercapnic and hypoxemic respiratory failure.

FBA continues to be a common reason for presentation to the emergency department, with a rate of 20.4 visits per 100,000 population in the USA (6). In a study by Saki et al, of 1,015 pediatric patients undergoing bronchoscopy for FBA, the location of the FB occurs in the right mainstem bronchus in 55% of patients, followed by left main bronchus, trachea, vocal cords, and less than 2% in both bronchi (7). As such, bilateral bronchial FBA are very rare. Mortality from FBA is low, cited at 0.42%, and most often related to complication with bronchoscopic retrieval of the object, such as hypoxic cardiac arrest and bronchial ruptures (8), although asphyxia at presentation does occur as well. Major complications occur in an estimated 1% of patients undergoing bronchoscopic removal of FB and include severe laryngeal edema, bronchospasm, pneumothorax, pneumomediastinum, cardiac arrest, tracheal or bronchial laceration, and hypoxic brain damage (8). Given the risk associated with bronchoscopy evaluation, ensuring patient optimization from a respiratory and hemodynamic standpoint is critical prior to the procedure.

While the indications for ECMO for cardiorespiratory support continue to expand in the critically ill population (9), the use of ECMO for FBA is rare, with only 57 pediatric patients described in the literature (1, 10). In a case series of 4 patients requiring ECMO for FBA by Anton-Martin et al, ECMO was used in 3 of 4 patients to attain stability prior to successful removal of the foreign body (1). Three of the patients had bronchoscopy attempted prior to cannulation but were unsuccessful for either technical reasons or patient decompensations, such as worsening hypoxemia. In a similar case by Alkhalifah et al, bronchoscopy could only be attempted after stabilization onto ECMO due to severe hypoxemia (10). In pediatric cases of airway trauma or congenital abnormalities, ECMO has been used as a bridge to surgical repair and complex tracheal reconstructions (11, 12). In adults, there has been an increase in the use of elective ECMO cannulations for surgical cases of distal airway obstruction such as tumors and other complex tracheal surgeries at high risk of airway blockade (13, 14). Elective cannulation to ECMO in pediatric patients has also been used successfully in cases of high-risk cardiac catheterization (15). With the expanding use of both elective and emergent ECMO in high risk patients, clinicians need to consider ECMO early in the patient's course, prior to deteriorations that lead to significant patient morbidity.

While elective cannulation to ECMO is increasing, pediatric patients on ECMO have a high risk of morbidity and mortality (16). In FBA cases requiring ECMO, the survival to hospital discharge is greater than 90%, while data on other complications are limited given there are only case reports described (1). In patients with near fatal asthma, an obstructive disease similar to FBA, who require ECMO support, the most common complications of ECMO are bleeding (28%), stroke (4.8%), acute kidney injury (25%) and need for renal replacement therapy (20%) (17). Our patient was diagnosed with an ischemic stroke during a surveillance MRI with no clinical features suspicious for strokes. Multiple etiologies could have contributed to the stroke: a thromboembolic process from ECMO, a rapid decrease in arterial CO_2_ after cannulation that might have contributed to increased cerebrovascular constriction and decreased perfusion, a phenomenon described in adult ECMO patients (18), or myocardial stun with resultant change in cardiac ejection and change in brain perfusion. This phenomenon of myocardial stun could be due to different factors and in our patient can be related to increase in oxygen free radicals (19). Additional explanations could include severe prolonged hypoxemia and/or hypoperfusion, however, our patient did not suffer from episodes of severe hypoxia, lowest saturations were transient decreases into the mid 70%, and there were no hypotensive episodes recorded. Ideal timing of ECMO cannulation is yet to be determined, but data in myocarditis patients suggests that a shorter intubation to ECMO cannulation time prior to a cardiac arrest was associated with the best patient outcomes (20). Similarly, in pediatric patients with near fatal asthma, indications for ECMO cannulation included clinical deterioration despite optimal medical therapies, severe respiratory acidosis with pH 7.0–7.1 and PaCO2 90 mm-100 mm Hg, PaO2 <100 mm Hg, plateau pressure >30 cm water, and impending cardiac arrest (17). However, these are only recommended cut-offs, no published guidelines exist, and clinicians' perspective of patient trajectory is essential in the decision making. Given the complications of ECMO, compared with risk for cardiopulmonary collapse prior to, during, or after bronchoscopy evaluation, timing for initiation of ECMO is critical in the management of FBA.

In our patient, with the denied history of FBA by the family, the initial treatment of asthma was focused on bronchodilator and anti-inflammatory therapy to relieve bronchospasm and respiratory support with BiPAP to relieve severe respiratory distress, with response noted in the patient. This initial response in the first 48 h was likely due to reduction in the edema surrounding the foreign body or the dislodgement of the FB into a distal airway allowing transient clinical improvement. The deterioration after two days of therapy is unusual in status asthmaticus unless a complication has developed from therapy, for example, the development of an air leak, or the presence of an untreated infection. Intubation in FBA is often required to perform bronchoscopy (21), while intubation in severe asthma carries serious risks, such as pneumothorax and cardiac arrest (22, 23). General indications for intubation in status asthmaticus include severe hypoxia, altered level of consciousness, progressive exhaustion with CO_2_ retention and respiratory acidosis, and respiratory and cardiac arrest (24, 25). The decision to intubate our patient was based on impending respiratory failure presenting as bradypnea with CO_2_ retention despite medical management of bronchospasm and non-invasive therapy for respiratory distress support. Our team proceeded with this high-risk intubation in view of this deterioration on a high level of support with no additional therapies that can be offered. ECMO cannulation and perfusion team availability at the bedside was requested for the intubation to prevent prolonged time to cannulation if the patient deteriorated into cardiopulmonary arrest. If ECMO is available at the center where intubation is done, team notification might be prudent. In our case, the patient did not suffer an arrest, but the lung mechanics and degree of support prompted a semi-urgent cannulation. Diagnostic bronchoscopy was performed only once the patient was stable from a respiratory and hemodynamic perspective after mechanical ventilation and ECMO initiation.

## Conclusion

FBA is a common cause of pediatric respiratory distress, particularly in children under three years old, and can be a diagnostic challenge, especially if the presentation is atypical or the aspiration event is unwitnessed. This case demonstrates the need to revisit diagnoses in the lack of persistent and consistent clinical improvement as well as how ECMO can be utilised as a bridge to recovery and diagnosis, particularly given the risks of death and other severe complications associated with bronchoscopy in FB removal.

## Data Availability

The original contributions presented in the study are included in the article, further inquiries can be directed to the corresponding author.
